# Parent perspectives on the benefits and risks of child-livestock interactions

**DOI:** 10.3389/fpubh.2023.1050584

**Published:** 2023-01-27

**Authors:** Ryan T. Klataske, Trevor J. Durbin, Kathrine L. Barnes, Kyle Koshalek, Casper G. Bendixsen

**Affiliations:** ^1^Department of Sociology, Anthropology, and Social Work, Kansas State University, Manhattan, KS, United States; ^2^National Farm Medicine Center, Marshfield Clinic Research Institute, Marshfield, WI, United States

**Keywords:** health, safety, children, youth, livestock, risk, agriculture, anthropology

## Abstract

Growing up on a farm or ranch often involves interactions with livestock that present both potential risks and benefits to children. While these “child-livestock interactions” contribute to the burden of agriculturally related injuries to youth in the United States, they may also result in improved immunological health and other benefits. Agricultural upbringings are also widely perceived to improve physical, cognitive, and skill development of children, contributing to a combination of potential benefits and risks known as the “farm kid paradox.” Although previous studies show the health impacts of child-livestock interactions, less is known about the ways in which farm and ranch parents perceive the benefits and risks of these interactions, and how and why they choose to raise children around livestock. Our research addresses this gap by analyzing data from semi-structured interviews with 30 parents of children between the ages of 10–18 who produce beef cattle in Kansas. This research is part of a larger anthropological study of the benefits and risks of child-livestock interactions involving parents on beef and dairy operations in multiple states, along with agricultural safety and health professionals. The results offer insights into the experiences, practices, and perspectives of parents, outlining agricultural ways of life in which safety and relations to risk are shaped by patterns of production, family dynamics, values and habits, and other social and cultural dimensions. These insights deepen our understanding of parents' perceptions of both benefits and risks of agricultural childhoods.

## 1. Introduction

Growing up around livestock on a farm or ranch comes with both benefits and risks—a combination Bendixsen (1) has called the farm kid paradox. The paradox is that “certain aspects of farm life that expose children to harm are also linked to positive health outcomes.” Rural upbringings may produce various physical, cognitive, and skill development benefits including a strong work ethic and problem-solving abilities. Recent research suggests measurable immunological benefits as well (2–17). Yet, growing up on a farm or ranch also involves risks to safety and health from interactions with livestock, machinery, chemicals, and other aspects of home environments that are also often workplaces, involving both work and play (18, 19). According to the National Farm Medicine Center, a child dies in an agriculture-related incident about once every 3 days and thousands of children are injured each year (20). Keeping children alive and thriving in vibrant rural communities is a common goal for both parents and advocates of agricultural safety and health. Supporting and effectively communicating with parents requires a deeper understanding of their needs, constraints, values, and lives. So, for those who want to see children continue to grow up on farms and ranches—and who also want to see fewer debilitating injuries and death among children—the question of how parents perceive benefits and risks is important.

In our research, we have explored how farm and ranch parents think about the benefits and risks of raising children around livestock, why they think it is important, how they make decisions, and other questions designed to understand their perspectives and experiences. What is it like to raise kids and livestock at the same time, and what do parents have to say about how to do it safely?

Our work builds on previous biomedical research on respiratory and immunological health impacts of childhood livestock exposure as noted above, anthropological research exploring questions about how we think and talk about occupational risk (21, 22), translational research on tailoring safety and health information (23–26), along with literature on the relevance and importance of culture in agricultural safety and health research (1, 27–31). We also use the Socio-Ecological Model (SEM) as a conceptual framework for thinking about the embeddedness of farm and ranch families in broader levels of social organization. This model helped to guide research design by focusing our attention not only on parents and their children, but also agricultural professionals and organizations at multiple levels. It complements our anthropological understanding of the complex ecological, social, and cultural dynamics of human behavior and reminds us to explore potential opportunities for interventions at multiple scales (32).

## 2. Design and methods

In 2021 and 2022, we conducted semi-structured interviews (33) with 30 beef producing farm and ranch parents in Kansas, as well as a free listing activity (33, 34) with 10 of these participants. In this activity, we had them describe child-livestock interactions by listing activities (both work and play) that their children perform with cattle, along with perceived benefits and potential risks. This research is part of a larger anthropological study that also involves dairy-operating parents in multiple states, along with agricultural health and safety professionals and others related to youth and livestock. In addition to visiting farms and ranches, we also utilized rapid ethnographic assessment techniques (35) to observe child-livestock interactions and interact with both parents and professionals in the context of county fairs in Kansas and Nebraska.

We recruited research participants through a Kansas State University extension network, at a local county fair, and through personal connections in rural communities surrounding Manhattan, Kansas. This recruitment process and the research, in general, were shaped and strengthened by our team members' rural and agricultural backgrounds and experiences, which inform our understanding of what it is like to grow up on a farm, ranch, or in a rural community.

Interviews took place in participants' homes, on their farms or ranchland, at workplaces, in public spaces, and online *via* videoconferencing due to the constraints of COVID-19. Each interview typically lasted around 45–60 min and sometimes involved additional conversation and tours of farms and livestock. We recorded field notes during visits and created written lists with some participants to visualize and engage in the free-listing activity together. Participants received informational handouts about the project and provided signed informed consent prior to interviews, which were recorded and transcribed. They were offered and later mailed compensation for completing the interviews as a gesture of appreciation.

Our analysis in this report is based on the narratives and perspectives of parents collected through semi-structured interviews and their discussions of perceived benefits and risks during the free listing activity. The data from free listing, however, will be subjected to more formal qualitative and quantitative analysis involving pile sorting in later stages of our project. We analyzed and interpreted the resulting data through an iterative process of immersion and crystallization (36) involving repeated team-based discussions and triangulation, along with feedback from external scientific advisors. Based on the qualitative data analysis process outlined by Babchuk (37), our approach also involved the identification and interpretation of multiple themes and patterns emerging from memoing, open coding, the categorization of codes, and the generation of themes from categories. We critically examined and discussed these interpretations as a team and invited feedback from peers and parents associated with agriculture.

## 3. Findings

### 3.1. Parents produce a continuum of child-livestock interactions

The Kansas farm and ranch parents we met in this study all raise children and cattle. They are all beef producers—with at least one head of cattle—and at least one kid between the ages of 10–18. We prioritized families that lived on farms and ranches, many of which turned out to be relatively diversified operations involving cattle, horses, pigs, goats, sheep, poultry, hay, and other crops and animals. Many of the children in these families raise and care for 4-H or FFA show animals and often form social networks with other farm families. The families consisted of two to five children, with an average of around three (2.9) children. Operations ranged in size from small-scale “hobby farms” (< 20 acres) to much larger-scale commercial livestock and crop production (around 4,000 acres). Most families live in the eastern half of Kansas and many own and/or rent land in the Flint Hills, a unique tallgrass prairie ecoregion with a mosaic of grasslands, woodlands, streams, hills, and both grazing and cropland. Importantly, the beef producing parents in our study are not agricultural workers hired by farms and ranches, who experience different levels of agency and precarity. We are also not focused on parents whose children are hired employees on others' agricultural operations.

On one end of the spectrum, we met a family living on 20 acres on the outskirts of Kansas City, a major urban center, with one cow-calf pair, alpacas, chickens, and rabbits for 4-H, where the father worked off the farm and the mother home-schooled their daughter, who cared for show animals and played in the woods on the property. Another similar family lived on < 10 acres outside Manhattan, with several horses and cattle for show in 4-H. On the other end of the spectrum, we met parents involved in multi-generational family farms and ranches, often with multiple properties, interests, and areas of commercial operation. We interviewed, for example, a full-time cow-calf producer raising four children on 3,000 acres in the Flint Hills, specializing in high-value breeding and genetics. We also spoke with a mother whose husband and four children live on a 360-acre diversified farm and work on their family's multi-generational, 4,000-acre commercial crop and cattle operation. In general, we met parents raising children on farms and ranches of varying sizes, interacting with cattle and often an assortment of other animals for show, personal consumption, commercial production, and a variety of other reasons including personal enjoyment, hobbies, work, and learning opportunities. We met beginning farmers with off-farm employment and full-time farmers and ranchers with agricultural backgrounds and livelihoods—and a range of people in between.

We found that this spectrum of farm and ranch operations produces a continuum of child-livestock interactions. While some children are primarily involved in the care of show or hobby animals, others are much more integrated into the economic system of family farms and ranches. Some parents described the important contributions and intricate involvement of their children in the work of livestock production and agricultural operations. These tasks included cattle handling and processing, along with the use of horses, equipment, vehicles, and other machinery. In some families, children played an active role in the division of labor and systems of production on family farms and ranches.

### 3.2. Parents share similar motivations

While parents differed in the extent to which they involve children in livestock production and other aspects of farm and ranch work, they expressed similar motivations and a common intentionality in their decisions to raise their children around livestock. Whether they chose to raise children on a small-scale, less commercially oriented farm, or they continue a family tradition of agricultural production, parents described their lifestyle as an intentional decision to provide children with opportunities and benefits associated with farm and ranch life. As one couple explained, raising their four children on 23 acres of hilly pasture with “a handful of cows,” two dozen goats, rabbits, chickens, ducks, dogs, and cats provides opportunities to work and have responsibilities, to learn about biology and animal husbandry, and to connect with their land and livelihood.

We also found that parents are motivated by a shared desire to cultivate certain kinds of learning opportunities and raise children to become certain kinds of people. Parents expressed value in teaching and learning through work, hands-on experience, interactions with animals, and some exposure to risk and challenges. They also discussed the importance of connections to food systems, land, and non-human species. These transformational experiences are envisioned as a foundation for producing people with a strong work ethic, practical skills, self-reliance, responsibility, and other traits conducive to the continuation of an agricultural way of life and other forms of demanding labor in our modern world. Child-livestock interactions are viewed by parents as part of this process of preparing children to survive, thrive, and contribute to society. As one father exclaimed, “We want to make dang sure that they are a good person in society.”

### 3.3. Parents perceive a multitude of benefits

In our interviews, parents described a multitude of reasons why raising children around livestock is important, listing the many benefits that these interactions provide. Parents perceived this intertwined upbringing to be important because it introduces and prepares children for agricultural work and ways of making a living, promotes an active outdoor lifestyle, develops valuable skills and capabilities, fosters connections to food and places, passes along tradition, and instills values and character. Some of the other benefits of growing up around livestock that were commonly listed by parents include:

- Responsibility.
- Work ethic.
- Discipline, persistence, and delayed gratification.
- Knowledge of life cycles, life and death, biology, and science.
- Understanding of loss.
- Decision-making abilities and time management.
- Public speaking abilities (from showing and speaking to judges and other adults).
- Ability to deal with loss, failure, and disappointment.
- Situational awareness.
- Attention to detail.
- Self-reliance and independence.
- Ability to use and care for machinery and tools, including horses.
- Animal handling skills and knowledge of animal behavior.
- Exposure to animals and compassionate relationships with animals.
- Physical strength.
- Fun and family togetherness.
- Access to the outdoors and rural environments.
- Financial management and math skills.
- Problem-solving skills.
- Pride, confidence, and a sense of accomplishment.
- Exposure to diverse experiences.

Parents often described raising children and livestock at the same time as rewarding. Of course, some parents discussed the challenges of keeping children safe, motivating them to do chores, and balancing farm life with the demands of school, sports, and other activities. Despite the challenges of parenting on farms and ranches, we heard often about a multitude of benefits and the pride parents felt in the quality of people their kids were becoming.

### 3.4. Parents believe benefits outweigh potential risks

In addition to benefits, parents listed various potential risks associated with raising children around livestock. While some parents perceived a low level of risk to their childrens' safety and health, most parents easily identified and described in detail the risks of livestock handling, horseback riding, large and unpredictable animals, the use of tools, equipment, and machinery, and other aspects of farm and ranch life. These included risks, for example, of smashed fingers and toes, kicks from livestock, trampling, and injuries from horse or vehicle accidents. Some parents discussed their worries of potentially serious injuries and concerns about safety, but the most common theme we heard is that the benefits of growing up around livestock on a farm or ranch outweigh the risks.

As one mother told us, raising children around livestock is “a balance.” This idea of balancing benefits and risks is a common way that parents talked about the challenge of providing learning opportunities with enough exposure to risk to produce benefits, while avoiding severe injury or death. She explained that, as parents, they “recognize those risks but understand that, at least in our minds, those benefits that the kids are gonna get outweigh those risks.” The objective then, as other parents discussed as well, is to “manage those risks so the benefits outweigh it.”

To strike this balance and manage risk, parents described a range of decision-making strategies including assessing age and strength, incremental responsibility and exposure, hands-on mentoring, role-modeling, and monitoring. For some parents, decisions about the activities children perform on the farm or ranch are at least partially determined by what needs to get done. The common narrative, however, was that the benefits of child-livestock interactions outweigh potential risks.

### 3.5. Parents see limited exposure to risk as a benefit

Another key reason why farm and ranch parents believe benefits outweigh potential risks is that, not only is exposure to risk an important part of learning, but it is also one of the benefits of growing up on a farm or ranch. Exposure to the various risks, challenges, and occasional failures associated with caring for livestock is seen by many parents as beneficial for the development of the skills, characteristics, and resiliency that they value and strive to cultivate in their children. Consequently, we did not encounter parents who prioritized eliminating risk or preventing all injuries. Instead, we heard that some injuries are expected and acceptable, and sometimes opportunities to learn and improve. Exposure to risk and some level of injury, we heard, might make children safer.

As one parent told us, her children might be safer “because they're exposed to more.” Others, for example, expressed beliefs that their kids' exposure to farm vehicles will make them safer drivers, and that limited and controlled exposure to livestock at a young age will make them safer, more aware, and more capable of handling risks of livestock in the future.

One rancher we met told his children who show cattle that, “You're gonna get stepped on. You're gonna get kicked. It's just part of it.”

Another mother recalled how her son gained a “healthy fear” and learned a valuable lesson about attention, respect, and cattle handling after a kick by a calf. Her story served to illustrate how exposure to risk can be seen as a benefit in certain situations, and how benefits can be seen to outweigh risks. This incident, in which her son was kicked but not severely injured, was “a learning experience,” she explained. “It's just like with anything, there are risks with it but the experiences and the knowledge that they've gained from having these animals, to us, most definitely outweighs [the risk].”

## 4. Discussion and conclusion

Our findings suggest that farm and ranch parents in Kansas are not naïve to the idea that raising children around livestock inherently involves a combination of benefits and risks, or that certain aspects of farm life expose children to harm while also producing positive outcomes. Instead, parents described this combination in rich detail as they discussed the ways their children interact with cattle and take part in everyday farm or ranch life. Parents articulated the value of child-livestock interactions and the intentionality of an agricultural way of life that produces diverse opportunities to learn, grow, and become valuable members of society.

As one parent noted, family farms are seen as environments “naturally conducive” to exposing children to experiences that develop valuable skills and instill certain qualities and characteristics. Exposure to risk and even some level of injury is accepted and, in fact, valued as a pedagogical approach and parenting philosophy. Parents talked about balancing or calculating risk, and they expressed awareness of various risks to their children performing a wide array of tasks. Many of the parents we interviewed also viewed exposure to risk as a benefit—sometimes even as a sort of gift shared among parents, children, and animals. This means that benefits and risks are not necessarily dichotomous, but rather much more complex and interwoven in the minds and lives of farm and ranch families.

In other words, some exposure to risk, challenging situations, and diverse experiences—along with some level of non-fatal and non-debilitating injury—is actually seen as a benefit since it entails vital opportunities to learn, develop, and perhaps become safer in the long-term. This perspective is part of a shared vision of the kinds of humans that parents are trying to raise—ones with character and a strong work ethic, practical and valuable skill sets, self-reliance, responsibility, sound judgment, and other traits needed to sustain agricultural heritage and ways of life, or to survive and thrive in other demanding jobs and precarious times. Perhaps most importantly, parents believe that the benefits of child-livestock interactions outweigh potential risks.

Understanding how farm and ranch parents perceive the benefits and risks of child-livestock interactions may help to bridge cultural and communication gaps between rural communities and agricultural safety and health professionals. This knowledge and cultural competence may also improve the design, trustworthiness, and adoptability of injury prevention messaging and intervention strategies, including work guidelines and other recommendations for parents (38–40). One of the strengths of our anthropological approach is that it focuses on child-livestock interactions from the point of view of parents, but our findings are also limited to parent perspectives and experiences, as opposed to those of children. The amount of time we spent on farms and ranches was also limited, constraining opportunities for observations and participation in ordinary family life.

Nevertheless, to translate injury prevention materials and make agricultural health and safety resources more meaningful for parents, our work suggests that because parents emphasize benefits, understanding and incorporating the perceived benefits of child-livestock interactions may be key. Focusing only on potential risks—or on the elimination of all possible opportunities for injury—may alienate farm and ranch parents by obscuring how and why they choose to raise children around livestock, and what doing so safely means to them. Acknowledging the multitude of perceived benefits that come from working and growing up on a farm or ranch is one plausible step toward more effective and culturally sensitive communication with parents and rural communities.

A takeaway from this research is that for ideas about how to prevent debilitating injuries and death of children on farms and ranches to reach and impact parents, it may be essential to recognize how they perceive and balance both the benefits and risks of child-livestock interactions, and why they desire and cultivate an agricultural childhood for their families. Supporting parents in their efforts to raise both healthy children and livestock at the same time may also involve envisioning the kinds of humans and society they seek to shape.

## Data availability statement

The raw data supporting the conclusions of this article will be made available by the authors, without undue reservation.

## Ethics statement

This study involving human participants was reviewed and approved by Kansas State University Institutional Review Board. The patients/participants provided their written informed consent to participate in this study.

## Author contributions

RK conducted data collection and led latter stages of analysis and interpretation. RK and TD contributed to the conceptualization and writing of the article. All authors contributed to the production of this manuscript, research design and methodology, data analysis and interpretation, review, editing, preparation, and approval of the submitted manuscript.
